# Association between hospitalizations for sensitive conditions and quality of primary care

**DOI:** 10.11606/s1518-8787.2023057004879

**Published:** 2023-10-30

**Authors:** Filipe Malta dos Santos, César Macieira, Antônio Thomaz Gonzaga da Matta Machado, Elis Mina Seraya Borde, Alzira de Oliveira Jorge, Bruno Abreu Gomes, Alaneir Fatima dos Santos

**Affiliations:** I Universidade Federal de Minas Gerais Faculdade de Medicina Programa de Pós-graduação em Saúde Pública Belo Horizonte MG Brasil Universidade Federal de Minas Gerais . Faculdade de Medicina . Programa de Pós-graduação em Saúde Pública . Belo Horizonte , MG , Brasil; II Universidade Federal de Minas Gerais Faculdade de Medicina Núcleo de Educação em Saúde Coletiva Belo Horizonte MG Brasil Universidade Federal de Minas Gerais . Faculdade de Medicina . Núcleo de Educação em Saúde Coletiva . Belo Horizonte , MG , Brasil; III Universidade Federal de Minas Gerais Faculdade de Medicina Belo Horizonte MG Brasil Universidade Federal de Minas Gerais . Faculdade de Medicina . Belo Horizonte , MG , Brasil

**Keywords:** Primary Health Care, Quality of Health Care, Family Health

## Abstract

**OBJECTIVE:**

To analyze the association between municipal rates of ambulatory care sensitive conditions (ACSC) hospitalization and the quality of primary health care (PHC), socioeconomic, and demographic variables and those related to local characteristics of the health system from 2010 to 2019.

**METHOD:**

Ecological time series study in Brazilian municipalities analyzing the correlation of ACSC hospitalization rates with PHC quality measured by the three cycles of the Primary Care Access and Program for improving primary care access and quality (PMAQ-AB). The study included municipalities whose teams participated in 80% or more of at least two PMAQ-AB cycles. The correlation between standardized ACSC hospitalization rates and PHC quality and other variables was analyzed. Spearman’s test was used between the response variable and numerical explanatory variables. Generalized equations estimation was used as a multivariate model associating ACSC hospitalization rates with the other variables over the years.

**RESULTS:**

A total of 3,500 municipalities were included in the models. The quality of PHC (PMAQ-AB score) showed an inverse association with the variation in ACSC hospitalization rates. Hospitalization rates fell by -2% per year every ten-point increase in the PMAQ-AB score, adjusted by the remaining variables. A one-unit increase in the beds per 1,000 inhabitants variable had an impact of approximately +6.4% on ACSC hospitalization rates. Regarding population size, larger municipalities had lower ACSC hospitalization rates. Increased PHC coverage and lower socioeconomic inequality were also associated with the reduction in hospitalizations.

**CONCLUSIONS:**

The reduction in ACSC hospitalization rates over time was associated with an increase in the quality of PHC. It was also associated with a reduction in the number of hospital beds and municipalities with better socioeconomic indicators.

## INTRODUCTION

Since 1994, when the Family Health Program as created, primary health care (PHC) in Brazil has expanded greatly throughout the country ^1^ . Since the creation of the Unified Health System (SUS), various government policies have sought to make PHC the system’s main gateway, as there is solid evidence that strengthening PHC lowers health costs, impacts on various indicators, and contributes to reducing health inequalities ^2^ .

Evaluating the expansion of PHC and improving its quality is essential for health planning in the country. One of the pillars of health quality assessment is the evaluation of results ^3^ . The indicator of hospitalizations for primary care-sensitive conditions is used in many countries as an instrument to evaluate their results ^4 - 6^ . Since the creation of the national list of hospitalizations for ambulatory care sensitive conditions (ACSC) in 2008, there has been an increase in studies on this subject in Brazil, seeking to understand its behavior and applicability ^7 , 8^ .

Time series of ACSC hospitalization showed a drop between 1999 and 2007 in all Brazilian regions and in the country as a whole. In general, an association was also found between this drop in hospitalizations and the expansion of PHC coverage in the country ^9^ . However, this association has not always proved to be true, especially in studies that analyze intra-urban or municipal data in isolation ^10 , 11^ . Other factors that also showed an association with ACSC hospitalization rates were socioeconomic conditions ^12 , 13^ , hospital beds available in the municipality ^11 , 14^ and health insurance coverage ^11^ . Some studies have tried to measure the correlation between PHC quality and ACSC hospitalization rates using the Primary Care Assessment Tool (PCATool) ^13 , 15^ or the Program for Improving Access and Quality in Primary Care (PMAQ-AB) ^12 , 16^ .

The PMAQ-AB is a nationwide program that began in 2011 and has been carried out over three cycles, the last one ending in 2019. Its main objective was to induce the expansion of access and improve the quality of PHC in the country, but it also fulfilled the role of evaluating family health teams through quality standards. PMAQ-AB stands out for its scope and capillarity, reaching around 39,000 teams and 96% of Brazilian municipalities in its last cycle ^17 , 18^ . It also stands out for maintaining aspects linked to: processes in the area of care, results in indicators and structures in basic units as the basis of its evaluation process, considering the context of Donabedian’s classic triad ^3^ .

However, there is still a lack of national studies evaluating the association between variation in ACSC hospitalization rates and PHC quality by municipality and its evolution over the years, as most studies only evaluate one municipality ^13 , 15^ or a single year ^12 , 16^ . Thus, the aim of this study was to analyze the association between municipal rates of ACSC hospitalization and quality of PHC, socioeconomic and demographic variables and those related to local characteristics of the health system between 2010 and 2019.

## METHODS

This work is characterized as an ecological time series study, in which correlations were analyzed between standardized ACSC hospitalization rates in Brazilian municipalities from 2010 to 2019 and the quality of care in PHC, characteristics of municipal healthcare, socioeconomic and demographic conditions.

Standardized ACSC hospitalization rates were calculated for each municipality using data from the *Sistema de Internação Hospitalar* (SIH – Hospital Admission System) from 2010 to 2019. Such rates were standardized by sex and age groups (0–4, 5–19, 20–59, 60–79 years), using as a reference the Brazilian population estimated by the Brazilian Institute of Geography and Statistics ( *Instituto Brasileiro de Geografia e Estatística* – IBGE) in 2014, as it was the midpoint of the period. The year of hospital discharge was considered to be the year of the patient’s hospitalization; and the municipality of residence defined the place where the hospitalization was counted ^9^ . Hospitalizations for diseases derived from the 10 ^th^ revision of the International Classification of Diseases (ICD-10) and validated as primary care-sensitive conditions in Brazil were considered, consisting of 20 diagnostic groups divided into 120 ICD-10 subgroups, excluding groups related to childbirth ^7^ . As recommended by the *Projeto ICSAP-Brasil* ^9^ , people aged 80 or over were also excluded from the study.

The study variables are presented in the Chart and include the quality of PHC; characteristics of local health systems such as: number of beds per 1,000 inhabitants, percentage of health insurance coverage and coverage of family health teams; socio-economic characteristics such as the Brazilian Deprivation Index ( *Índice Brasileiro de Privações* – IBP) and the Municipal Human Development Index ( *Índice de Desenvolvimento Humano Municipal* – MHDI), region and demographic characteristics such as sex and population size (small size I – up to 20,000 inhabitants; small size II – from 20,001 to 50,000 inhabitants; medium size – from 50,001 to 100,000 inhabitants; large size – from 100,001 to 900,000 inhabitants; metropolis – more than 900,000 inhabitants). The municipal size strata were defined according to a study by Castro et al. ^12^

**Chart:** Description of the study variables by type of variable, characteristics, data sources and year of database analysis.

Variable	Type of variable	Calculation procedures	Data source	Year of analysis of the base
Standardized ACSC hospitalization rate per 10,000 inhabitants	Response numeric variable	Annual ratio of the number of hospitalizations by ICD-10 of the ACSC hospitalization group to the estimated population of the municipality. Multiplied by 10,000 inhabitants and weighted by the Brazilian population in 2014.	SIH-SUS	2010 to 2019
Quality of PHC – score	Explanatory numeric variable	Simple mean of the scores of the teams from the same municipality, in the same cycle, in the PMAQ-AB certification process. Some municipalities show scores in only two cycles.	Database of the three PMAQ-AB cycles. Available on request from the Ministry of Health	2010–2012 (1 ^st^ cycle), 2013–2015 (2 ^nd^ cycle) e 2016–2019 (3 ^rd^ cycle)
Beds per 1,000 inhabitants	Explanatory numeric variable	Number of hospital beds agreed or contracted by the SUS, per 1,000 estimated resident inhabitants in a given municipality per year.	National Register of Health Establishments ( *Cadastro Nacional de Estabelecimentos de Saúde* – CNES)	2010–019
Percentage of health insurance coverage	Explanatory numeric variable	Ratio between the number of beneficiaries of private health insurance plans and the population of the estimated municipality multiplied by 100.	National Health Agency ( *Agência Nacional de Saúd* e – ANS), sector data and indicators	2010–2019
Percentage of FHT coverage	Explanatory numeric variable	Estimated population coverage of each municipality by family health teams and primary care teams calculated as a percentage.	e-Gestor AB Portal, Primary Care Information and Management ( *Secretaria de Atenção Primária à Saúde* )	2010–2019
Municipal size	Explanatory categorical variable	Classification of municipalities according to their population: small size I: up to 20,000 inhabitants, small size II: from 20,001–50,000 inhabitants, medium size: from 50,001–100,000 inhabitants, large size: from 100,001–900,000 inhabitants, metropolis: more than 900,000 inhabitants.	IBGE population estimate available on the Datasus website	2010–2019
Sex	Explanatory nominal categorical variable	Male and female according to the estimated population of each year.	IBGE population estimate available on the Datasus website	2010 to 2019
Region	Explanatory nominal categorical variable	Region of each municipality in the study.	IBGE data	2010 to 2019
MHDI	Explanatory numeric variable	National measure made up of three indicators: per capita income, schooling and life expectancy using data from the 2010 Census. The measure ranges from 0 to 1. The closer to 1, the better the human development.	Atlas of Human Development in Brazil	2010
IBP quintiles	Explanatory ordinal categorical variable	Composite measure of income, schooling, and household conditions, using data from the 2010 Census. A score was calculated for each Brazilian municipality and the standard deviation of the final score was used to group the municipalities into five ordinal categories, in which 5 was the index of greatest deprivation.	Cidacs website, Fiocruz(https://cidacs.bahia.fiocruz.br/IBP)	2010

ACSC: Ambulatory care sensitive conditions. ICD-10: International classification of diseases. PHC: Primary healthcare. SIH-SUS: Hospitalization System of the Unified Health System. PMAQ-AB: Program for improving primary care access and quality. MHDI: Municipal human development index. IBP: Brazilian deprivation index. FHT: Family Health Teams. IBGE: Instituto Brasileiro de Geografia e Estatística (Brazilian Institute of Geography and Statistics).

The main explanatory variable, PHC quality, was calculated using scores from the evaluation of the PMAQ-AB PHC teams, taken from the Program’s national database (Chart). The simple mean of the scores of family health teams from the same municipality received in each of the three PMAQ-AB cycles was calculated, which was different for each cycle. In the first and second cycles, the score prior to the certification process and the weighting by extracts were used to allow for better comparisons. In the third cycle, the score was multiplied by ten, since it was the only cycle in which the score varied from 0.0 to 10.0 and not from 0.0 to 100.0, thus allowing comparisons with the other cycles. In order to analyze the data, scores from the first PMAQ-AB cycle were repeated in 2010, 2011, and 2012 and correlated with the other variables or the same years; and so were those from the second cycle in 2013, 2014, and 2015 and those from the third cycle in 2016, 2017, 2018, and 2019 (Chart).

As an inclusion criterion for participation in the study, only municipalities that had at least two cycles with more than 80% of family health and primary care teams participating in the PMAQ-AB study were selected. A total of 3,800 municipalities were initially included in the study. The distribution pattern of ACSC hospitalization rates in these municipalities was checked and those which, in any of the years, had standardized rates lower than 15.0 or higher than 600.0 were excluded as they were discrepant values and could be related to errors in the information and surveillance system. As a result, another 280 municipalities were excluded from the analysis, leaving approximately 3,500 municipalities, with some variations from year to year, due to the creation of new municipalities in the period and missing data on hospitalization in a given year.

Considering that each municipality was in the ten years of analysis (2010 to 2019), and the stratification of rates by sex, there were around 70,000 units of analysis (3,500 municipalities* ten years * 2).

The distribution pattern of the variables was checked and, given that a non-normal pattern was observed, the relationship between the response variable and the numerical explanatory variables was verified using Spearman’s correlation ^19^ . IBP quintiles is an ordinal categorical variable, but for the purposes of analysis it was considered numerical.

In order to assess the influence of the variables on ACSC hospitalization rates over time, the generalized equations estimating (GEE) model was chosen ^20^ . Since the variables had a non-normal distribution, with continuous and positive values, the gamma pattern was assumed. Given that the correlations among the MHDI, IBP quintiles and percentage of health insurance coverage variables were strong, it was decided to run three different GEE models to avoid problems of multicollinearity.

To build the model, i.e., select the variables, the Stepwise method was used, which consists of adding (forward) and removing (backward) variables to define which ones will compose the final analysis ^21^ . In the forward step, the independent variables were univariately analyzed and those with a p-value < 0.20 were selected for the multivariate model. In the backward step, the variable with the highest p-value was removed from the analysis at a time, and the procedure was repeated until only significant variables remained in the final model. A 5% significance level was adopted for the multivariate model. All analyses were carried out in the R software, version 3.6.0, with the help of the geepack package.

The study was carried out in accordance with *Conselho Nacional de Saúde* (CNS – National Health Council) Resolution 466 of December 12, 2012. The SIH information is available on the Datasus website, without identifying patients.

## RESULTS

Table 1 shows the distribution pattern of the ACSC hospitalization rates and the other numerical variables over the ten years. It can be seen that the PHC quality variable showed the greatest variation in the units analyzed (5,670 to 7,004) due to the fact that some municipalities participated in only two PMAQ-AB cycles.

Mean hospitalization rates declined over the period, from 157.72 hospitalizations per 10,000 inhabitants in 2010 to 107.69/10,000 inhabitants in 2019. It is important to note that this is not the rate for Brazil, but a simple mean among the municipalities in the study. The mean score for the quality of PHC built fell from the first (58.95) to the second cycle (53.80) and the highest average was in the third cycle (59.55). The mean number of beds per 1,000 inhabitants also fell, from 1.58 in 2010 to 1.31 in 2019. The mean percentage of health insurance coverage remained between 7.67% and 9.40% and the mean percentage of FHT coverage remained between 0.85 and 0.91 (85% to 91%). It should also be noted that the data related to vulnerability indicators (MHDI, IBP quintiles) did not vary over the years as they are related to the 2010 Census ( Table 1 ).

When Spearman’s correlations between the ACSC hospitalization rates and the numerical variables year by year were made, a negative correlation was observed with the PHC quality indicator scores, with statistical significance in the respective municipalities, both in 2010 and in the years of the third cycle. In 2011 and 2012 there was no correlation and in the second cycle there was a positive correlation ( Table 2 ).

**Table 2:** Univariate correlations of ACSC hospitalization rates with numerical variables: PHC quality - score, MHDI, IBP quintiles, beds per 1,000 inhabitants, percentage of health insurance coverage, percentage of FHT coverage, according to Spearman’s test, correlation coefficient and p-value, in Brazilian municipalities, from 2010 to 2019.

Variables	Year	Quality of PHC – score ^a^	MHDI ^a^	IBP quintiles ^a^	Beds per 1,000 inhabitants ^a^	Percentage of health insurance coverage ^a^	Percentage of FHT coverage ^a^
Standardized ACSC hospitalization rate per 10,000 inhabitants	2010	-0.04 (0.008)	0.03 (0.007)	0.00 (0.794)	0.40 (< 0.001)	-0.03 (0.013)	-0.04 (0.001)
	2011	0 (0.744)	0.05 (< 0.001)	-0.01 (0.471)	0.37 (< 0.001)	-0.03 (0.021)	0.00 (0.741)
	2012	0.01 (0.324)	0.08 (< 0.001)	-0.04 (< 0.001)	0.34 (< 0.001)	0.00 (0.796)	-0.01 (0.527)
	2013	0.02 (0.042)	0.09 (< 0.001)	-0.05 (< 0.001)	0.34 (< 0.001)	0.00 (0.693)	-0.02 (0.192)
	2014	0.02 (0.07)	0.10 (< 0.001)	-0.08 (< 0.001)	0.33 (< 0.001)	0.03 (0.007)	-0.02 (0.096)
	2015	0.04 (< 0.001)	0.13 (< 0.001)	-0.10 (< 0.001)	0.34 (< 0.001)	0.05 (< 0.001)	-0.02 (0.161)
	2016	-0.04 (0.001)	0.15 (< 0.001)	-0.12 (< 0.001)	0.32 (< 0.001)	0.07 (< 0.001)	-0.03 (0.021)
	2017	-0.04 (0.001)	0.15 (< 0.001)	-0.12 (< 0.001)	0.34 (< 0.001)	0.07 (< 0.001)	-0.02 (0.179)
	2018	-0.04 (0.001)	0.14 (< 0.001)	-0.11 (< 0.001)	0.32 (< 0.001)	0.07 (< 0.001)	0.00 (0.896)
	2019	-0.04 (0.002)	0.13 (< 0.001)	-0.09 (< 0.001)	0.31 (< 0.001)	0.05 (< 0.001)	-0.01 (0.582)

ACSC: Ambulatory care sensitive conditions. PHC: Primary healthcare. MHDI: Municipal human development index. IBP: Brazilian deprivation index. FHT: Family health team.

^a^ Value of table cells: correlation coefficient and (p-value of coefficient).

The correlation of rates with socio-economic indicators (MHDI and IBP) year after year on a univariate basis showed that the highest hospitalization rates were in municipalities with best indices ( Table 2 ). Regarding the variables of municipal characteristics, the strongest positive association of ACSC hospitalization rates was observed with the variable beds per 1,000 inhabitants (0.31 to 0.40) ( Table 2 ).

The results of the final model, which correlated over the years using the GEE, are shown in Tables 3 and 4. In the univariate analysis ( Table 3 ), all the variables were significant (p-values < 0.05); it should be noted that the numerical variables that showed negative changes with the variation in hospitalization rates over the years, when increased by one unit were: quality of PHC – score, percentage of health insurance coverage and percentage of FHT coverage. The variables MHDI and IBP quintiles and beds per 1,000 inhabitants had positive changes.

**Table 3:** Univariate associations of standardized ACSC hospitalization rates per 10,000 inhabitants along with the study variables: quality of PHC - score, MHDI, IBP quintiles, percentage of health insurance coverage, beds per 1,000 inhabitants, percentage of FHT coverage, municipal size, region, sex, all in Brazilian municipalities, from 2010 to 2019.

Variables	Univariate analysis			

	β	Exp (β)	Alteration	p-value
Quality of PHC – score	-0.002	0.998	-0.21%	< 0.001
MHDI (:100) ^a^	0.393	1.482	0.48% ^a^	0.005
IBP quintiles	0.018	1.018	1.81%	0.016
log (Percentage of health insurance coverage) ^b^	-0.097	0.907	-9.26%	< 0.001
Beds per 1,000 inhabitants	0.069	1.071	7.15%	< 0.001
Percentage of FHT coverage	-0.131	0.877	-12.27%	< 0.001
Municipal size				
Small I	-	1	-	-
Small II	0.013	1.013	1.34%	0.573
Medium-sized	-0.189	0.828	-17.23%	< 0.001
Large	-0.425	0.654	-34.64%	< 0.001
Metropolis	-0.321	0.725	-27.48%	< 0.001
Region				
North	-	1	-	-
Southeast	-0.008	0.992	-0.83%	0.86
Midwest	-0.196	0.822	-17.76%	< 0.001
Northeast	-0.13	0.878	-12.19%	0.002
South	0.056	1.057	5.71%	0.174
Sex				
Female	-	1	-	-
Male	-0.126	0.881	-11.86%	< 0.001

PHC: Primary healthcare. MHDI: Municipal human development index. IBP: Brazilian deprivation index. FHT: Family health team. β: coefficient β.

^a^ The alteration was divided by 100 to take into account an alteration of 0.01 in the MHDI variable.

^b^ Percentage values for health insurance coverage were evaluated by log and 0.01 was added to all percentages because many units of analysis has a value of 0.

In the multivariate analysis, in the first model, using the variables in Table 1 (except for the IBP quintiles and the percentage of health insurance coverage), it was observed that municipal hospitalization rates for ACSC hospitalization declined as the quality of PHC improved over the years, and it was found that for every 10-point increase in the quality score, there was a -2.10% drop in the annual hospitalization rate; the 1% increase in FHT coverage reduced hospitalization by -11.81% each year. Increasing the MHDI by 0.10 resulted in a reduction in hospitalization rates of -4.90%. On the other hand, increasing the beds per 1,000 inhabitants variable resulted in an increase in hospitalization rates for primary care-sensitive conditions ( Table 4 ).

**Table 4:** Multivariate associations of standardized ACSC hospitalization rates per 10,000 inhabitants with the study variables in three models, according to the β coefficient, Exp(β), percentage alteration in the case of a one-unit increase in the numerical variable, p-value of the coefficient and Brazilian municipalities, between 2010 and 2019.

Variables	Model 1 - multivariate with MHDI ^c^				Model 2 - multivariate with IBP quintiles ^d^				Model 3 - multivariate log (Percentage of health insurance coverage) ^e^			
		
	β	Exp (β)	Alteration	p-value	β	Exp (β)	Alteration	p-value	β	Exp (β)	Alteration	p-value
Quality of PHC – score	-0.002	0.998	-0.21%	< 0.001	-0.002	0.998	-0.21%	< 0.001	-0.002	0.998	-0.20%	< 0.001
MHDI (:100) ^a^	-0.668	0.513	-0.49% ^a^	0.001	-	-	-	-	-	-	-	-
IBP quintiles	-	-	-	-	0.076	1.079	7.90%	< 0.001	-	-	-	-
log (Percentage of health insurance coverage) ^b^	-	-	-	-	-	-	-	-	-0,111	0.895	-10.47%	< 0.001
Beds per 1,000 inhabitants	0.062	1.064	6.0%	< 0.001	0.062	1.064	6.39%	< 0.001	0.062	1.064	6.42%	< 0.001
Percentage of FHT coverage	-0.126	0.882	-11.81%	< 0.001	-0.132	0.877	-12.34%	< 0.001	-0.123	0.884	-11.59%	< 0.001
Municipal size												
Small I	-	1	-	-	-	1	-	-	-	1	-	-
Small II	0.039	1.04	4.02%	0.106	0.046	1.048	4.75%	0.059	0.075	1.078	7.77%	0.004
Medium-sized	-0.132	0.876	-12.38%	< 0.001	-0.108	0.897	-10.28%	< 0.001	-0.043	0.958	-4.23%	0.157
Large	-0.403	0.668	-33.18%	< 0.001	-0.364	0.695	-30.48%	< 0.001	-0.281	0.755	-24.48%	< 0.001
Metropolis	-0.338	0.714	-28.65%	< 0.001	-0.291	0.748	-25.24%	< 0.001	-0.189	0.828	-17.19%	< 0.001
Region												
North	-	1	-	-	-	1	-	-	-	1	-	-
Southeast	0.001	1.001	0.10%	0.983	0.016	1.016	1.57%	0.73	0.116	1.123	12.33%	0.015
Midwest	-0.208	0.812	-18.80%	< 0.001	-0.195	0.823	-17.75%	< 0.001	-0.185	0.831	-16.89%	< 0.001
Northeast	-0.051	0.95	-5.01%	0.2	0.004	1.004	0.35%	0.93	0.116	1.123	12.28%	0.007
South	0.113	1.119	11.91%	0.008	0.165	1.18	17.99%	< 0.001	0.243	1.275	27.51%	< 0.001
Sex												
Female	-	1	-	-	-	1	-	-	-	1	-	-
Male	-0.117	0.89	-11.05%	< 0.001	-0.117	0.889	-11.05%	< 0.001	-0.123	0.884	-11.56%	< 0.001

PHC: primary healthcare. MHDI: municipal human development index. IBP: Brazilian deprivation index. FHT: family heath team.

^a^ The alteration was divided by 100 to consider an change of 0.01 in the MHDI variable.

^b^ The percentage of health insurance coverage assessed by log and 0.01 was added to all values because many units of analysis had a value of 0.

^c^ 1st model with the MHDI variable and without IBP quintiles and percentage of health insurance coverage.

^d^ 2nd model with IBP quintiles and without MHDI and percentage of health insurance coverage.

^e^ 3rd model with percentage of health insurance coverage and without MHDI and IBP quintiles.

In the second model, including the IBP quintiles variable and excluding the MHDI and percentage of health insurance coverage, the IBP showed a positive change. In municipalities with higher vulnerability quintiles, there was a positive change in hospitalization rates, while values of the other variables remained the same ( Table 4 ).

In the third multivariate model, excluding MHDI and also IBP quintiles, and using the percentage of health insurance coverage, the association found with health insurance coverage was -11.06% in rates for each 1% increase in coverage, adjusted for the variables in the model ( Table 4 ). The other numerical variables in models 2 and 3 kept their associations with hospitalization rates similar to model 1.

Among the categorical variables, negative associations were found with medium, large, and metropolitan areas, when compared to small I municipalities, while the other variables remained fixed in all models ( Table 4 ).

In terms of region, the number of hospitalization rates in the Midwest had a negative variation compared to the North in all three models of around -17%. In the South, hospitalization rates for ACSC hospitalization were higher than in the North, ranging from 11.91% to 27.51% in the study models. In the Southeast and Northeast there was a positive association only in model 3. Regarding sex, male hospitalization rates were lower than female hospitalization rates by -11.05% in all models ( Table 4 ).

## DISCUSSION

The study showed a downward trend in the mean of standardized ACSC hospitalization rates over the ten-year period, corroborating the findings of time series studies in previous periods ^9 , 11^ . Three models were used in the multivariate analysis and factors that showed an association with the reduction in hospitalizations were: improvement in the quality of PHC (as measured by PMAQ scores), increased coverage of the family health teams (FHT), reduction in the number of hospital beds linked to the SUS and increased coverage of health plans. In addition, municipalities with better socioeconomic indicators (measured by the IBP and MHDI), medium-sized, large, and metropolitan municipalities, living in the Midwest region and being male were associated with lower rates, while an increase in hospitalization rates was observed as the number of beds per inhabitant got bigger.

The study found an association between lower ACSC hospitalization rates in municipalities with better performance in the PMAQ-AB. These results confirm previous studies which showed a reduction in ACSC hospitalization rates when evaluating the first two cycles of PMAQ-AB ^12 , 16^ , the increase in the number of teams joining over the three cycles ^22^ , or which compared municipalities that joined PMAQ-AB with those that did not ^23^ . This study advances by indicating that, over a ten-year period, even among municipalities that participated with at least 80% of their teams, a ten-point increase in scores over the course of the PMAQ-AB cycles is associated with a reduction in rates of -2% per year, adjusted for other variables.

However, the association with PHC quality was not found by Gonçalves et al. ^15^ in a cohort study in Porto Alegre, nor by Mendonça et al. ^13^ in a study in Belo Horizonte. Both studies used the PCATool as a quality indicator and evaluated time series in a single municipality.

When relating the fall in ACSC hospitalization rates to FHT coverage, municipalities that had a 1% increase in the indices showed reductions in the rates for the period by -11.5 to -12.5%. This correlation was also observed in previous studies, which considered that increased FHT coverage would be an explanatory factor for this improvement ^9 , 24^ .

The univariate analysis over a one-year period showed that higher FHT coverage rates were not associated with lower hospitalization rates. This differs from the increase in coverage over the years, as this movement may be related to a real increase in investment, strengthening and expansion of PHC ^11 , 25^ .

Regarding socio-economic variables, it was found that an increase in the MHDI, associated with better living conditions, resulted in a drop in hospitalization rates. The IBP also identified a correlation in the quintiles of greater vulnerability, greater poverty, and increased hospitalization. These findings are in line with other studies that correlated vulnerability and hospitalization and that places with greater deprivation have worse health indicators ^12 , 13 , 26^ .

The variable beds per 1,000 inhabitants showed the highest correlation in all years in the univariate analysis and maintained a positive association in the three multivariate models investigated. The increase in the beds per 1,000 inhabitants’ indicator affected the growth in ACSC hospitalization rates, adjusted for the other variables. This association has already been found in other studies and may be related to the greater demand for hospitalization in places with more beds ^12 , 23 , 27 , 28^ .

The study found that the increase in health insurance coverage resulted in a drop in ACSC hospitalization. The findings of a reduction in ACSC hospitalizations related to an increase in private health insurance coverage have also been described ^12^ . It is possible that this indicator reflects better socio-economic conditions in these municipalities. It may also be related to the absence of hospitalization data in the non-SUS sector when calculating ACSC hospitalization rates.

Regarding demographic characteristics, in all three models there was a decrease in rates for medium, large-sized municipalities and metropolitan areas compared to small-sized municipalities. This finding is consistent with results of the study by Carmo ^28^ (2016), which pointed out that hospitals with up to 49 beds, considered small, accounted for 35% of the country’s ACSCs hospitalizations in 2015. The author stated that this profile results from the low complexity of hospitals, little diversity in the specialties offered, and that, in the state of Minas Gerais, almost half of them were located in cities of up to 10,000 inhabitants and such hospitals present greater demand and pressure for hospitalization ^3 , 28^ .

Men have lower hospitalization rates. Studies show that women generally have a better perception of the signs and symptoms of illnesses and seek health services more often ^29^ , which can result in greater opportunities for treatment and hospitalization.

These findings are important to reinforce the need to invest in PHC quality and evaluation programs, given the discontinuity of the PMAQ-AB. It is suggested that new follow-up studies be carried out in places that have invested in PHC quality, as well as studying the impact of variation over time in socio-economic conditions. Studies should also investigate the correlation between smaller municipalities and greater demand for hospitalization in these locations.

### Limitations

This is an ecological study and may contain association biases due to the concurrence of many interrelated explanatory factors. The use of ACSC hospitalization rates requires careful interpretation, as they may not only reflect PHC performance. This is an indicator that relates to the organization of health systems and is influenced by various other factors ^11^ . There may also be problems related to the hospitalization database because it does not include data from hospitals not affiliated with the SUS, which would interfere with the analysis and interpretation, especially in cities presenting greater health insurance coverage ^12 , 23^ . Even so, the SIH is a significant, reliable source of information, enabling relevant epidemiological analyses of hospital morbidity ^9^ . The IBP and MHDI variables are based on the 2010 Census and cannot measure the socioeconomic variation of municipalities over the period, but they remained in the model as they measure social inequalities. It should also be noted that adherence to the PMAQ-AB by municipalities and teams to be evaluated was voluntary, so there may be a bias in the selection of teams, especially in the first cycle.

## CONCLUSIONS

The study showed that better results in the quality of PHC in Brazil resulted in a reduction in ACSC hospitalization rates, reaffirming the need for progress in the country beyond the expansion of primary care coverage. It was also observed that places with greater socioeconomic deprivation had higher hospitalization rates, confirming that social inequality has an important relationship with health outcomes. The availability of beds per 1,000 inhabitants, especially in municipalities with fewer than 20,000 inhabitants, seems to be related to more hospitalizations.

The study also revealed the need to invest in evaluative programs considering the Donabedian triad on a national scale that can play a role in inducing improvements in PHC and indicating which directions to take towards a more effective and resolutive health system.

**Table 1:** Description of the distribution pattern of ACSC hospitalization rates and numerical variables: quality of PHC – score, beds per 1,000 inhabitants, percentage of health insurance coverage, percentage of FHT coverage, MHDI, IBP quintiles, by number of units of analysis, mean, standard error, 1st, 2nd, 3rd quartile, and p-value, in Brazilian municipalities, in the period 2010–2019.

Variables	Year	Valid nº	Mean	SE	Q1	Q2	Q3	p-value
Standardized ACSC hospitalization rate per 10,000 inhabitants	2010	6,938	157.72	1.21	82.22	132.48	208.59	< 0.001
	2011	6,968	144.35	1.12	76.18	119.72	188.3	
	2012	6,908	135.19	1.07	70.47	111.25	177.79	
	2013	6,910	127.42	1.01	67.34	105.48	162.4	
	2014	6,948	126.05	1.03	65.07	101.69	161.08	
	2015	6,968	119.35	0.98	61.29	96.73	152.75	
	2016	6,992	114.08	0.95	58.89	91.06	145.11	
	2017	7,028	111.42	0.92	59.25	89.36	139.32	
	2018	6,984	109.9	0.9	58.44	88.96	137.44	
	2019	7,062	107.69	0.86	59.27	87.45	132.54	
Quality of PHC – score	2010	5,670	58.95	0.13	53.68	60.11	65.63	< 0.001
	2011	5,686	58.91	0.13	53.58	60.09	65.63	
	2012	5,644	58.99	0.13	53.71	60.13	65.63	
	2013	6,830	53.78	0.12	47.02	54.22	60.8	
	2014	6,870	53.86	0.12	47.15	54.25	60.92	
	2015	6,890	53.9	0.12	47.18	54.32	61.08	
	2016	6,934	59.55	0.14	52.17	60.28	67.2	
	2017	6,974	59.56	0.14	52.17	60.27	67.22	
	2018	6,932	59.54	0.14	52.17	60.3	67.22	
	2019	7,004	59.54	0.14	52.19	60.27	67.18	
MHDI	2010	6,938	0.67	0	0.61	0.67	0.72	1
	2011	6,968	0.67	0	0.61	0.67	0.72	
	2012	6,908	0.67	0	0.61	0.67	0.72	
	2013	6,910	0.67	0	0.61	0.67	0.72	
	2014	6,948	0.67	0	0.61	0.67	0.72	
	2015	6,968	0.67	0	0.61	0.67	0.72	
	2016	6,992	0.67	0	0.61	0.67	0.72	
	2017	7,028	0.67	0	0.61	0.67	0.72	
	2018	6,984	0.67	0	0.61	0.67	0.72	
	2019	7,062	0.67	0	0.61	0.67	0.72	
IBP quintiles	2010	6,938	4.01	0.01	3	4	5	0.999
	2011	6,968	4	0.01	3	4	5	
	2012	6,908	4	0.01	3	4	5	
	2013	6,910	4	0.01	3	4	5	
	2014	6,948	4	0.01	3	4	5	
	2015	6,968	3.99	0.01	3	4	5	
	2016	6,992	4	0.01	3	4	5	
	2017	7,028	4	0.01	3	4	5	
	2018	6,984	4	0.01	3	4	5	
	2019	7,062	4	0.01	3	4	5	
Beds per 1,000 inhabitants	2010	6,938	1.58	0.02	0	1.24	2.29	< 0.001
	2011	6,968	1.55	0.02	0	1.21	2.25	
	2012	6,908	1.53	0.02	0	1.18	2.21	
Beds per 1,000 inhabitants	2013	6,910	1.47	0.02	0	1.13	2.16	< 0.001
	2014	6,948	1.47	0.02	0	1.13	2.17	
	2015	6,968	1.45	0.02	0	1.09	2.15	
	2016	6,992	1.41	0.02	0	1.07	2.09	
	2017	7,028	1.4	0.02	0	1.04	2.06	
	2018	6,984	1.37	0.02	0	1.01	2.02	
	2019	7,062	1.31	0.02	0	0.94	1.95	
Percentage of health insurance coverage	2010	6,938	7.67	0.14	1.03	3.38	9.84	< 0.001
	2011	6,968	8.39	0.2	1.21	3.8	10.73	
	2012	6,908	8.85	0.25	1.36	4.06	11.32	
	2013	6,910	9.06	0.24	1.41	4.23	11.81	
	2014	6,948	9.44	0.18	1.6	4.65	12.84	
	2015	6,968	9.4	0.16	1.59	4.7	12.89	
	2016	6,992	9.11	0.15	1.54	4.64	12.54	
	2017	7,028	8.99	0.13	1.52	4.59	12.57	
	2018	6,984	8.93	0.13	1.55	4.68	12.62	
	2019	7,062	8.83	0.13	1.53	4.64	12.45	
Percentage of FHT coverage	2010	6,938	0.89	0	0.86	1	1	< 0.001
	2011	6,968	0.85	0	0.78	1	1	
	2012	6,908	0.85	0	0.78	1	1	
	2013	6,910	0.86	0	0.79	1	1	
	2014	6,948	0.89	0	0.87	1	1	
	2015	6,968	0.9	0	0.9	1	1	
	2016	6,992	0.91	0	0.93	1	1	
	2017	7,028	0.9	0	0.92	1	1	
	2018	6,984	0.9	0	0.9	1	1	
	2019	7,062	0.9	0	0.89	1	1	

ACSC: Ambulatory care sensitive conditions. PHC: Primary healthcare. MHDI: Municipal human development index. IBP: Brazilian deprivation index. FHT: Family health Teams. SE: Standard error. Q1: 1 ^st^ quartile. Q2: 2 ^nd^ quartile. Q3: 3 ^rd^ quartile
