# Supplementary material for: Association between hospitalizations for sensitive conditions and quality of primary care
Source: Rev Saude Publica. 2023 Oct 30;57:85. doi: 10.11606/s1518-8787.2023057004879 (PMC10631748; doi:10.11606/s1518-8787.2023057004879)
Supplement: Translation

# Translation: Associação entre internações por condições sensíveis e qualidade da atenção primária

**Authors:** Filipe Malta dos Santos, César Macieira, Antônio Thomaz Gonzaga da Matta Machado, Elis Mina Seraya Borde, Alzira de Oliveira Jorge, Bruno Abreu Gomes, Alaneir Fatima dos Santos

**Affiliations:** I Brasil Universidade Federal de Minas Gerais. Faculdade de Medicina. Programa de Pós-graduação em Saúde Pública. Belo Horizonte, MG, Brasil; II Brasil Universidade Federal de Minas Gerais. Faculdade de Medicina. Núcleo de Educação em Saúde Coletiva. Belo Horizonte, MG, Brasil; III Brasil Universidade Federal de Minas Gerais. Faculdade de Medicina. Belo Horizonte, MG, Brasil

**Keywords:** Atenção Primária à Saúde, Qualidade da Assistência à Saúde, Saúde da Família

## Abstract

**OBJETIVO:**

Analisar a associação entre taxas municipais de internações por condições sensíveis à atenção primária (ICSAP), com a qualidade da atenção primária à saúde (APS), variáveis socioeconômicas, demográficas e relacionadas a características locais do sistema de saúde, de 2010 a 2019.

**MÉTODOS:**

Estudo ecológico de séries temporais nos municípios brasileiros analisando a correlação das taxas de ICSAP, com a qualidade da APS medida pelos três ciclos do Programa de Melhoria do Acesso e da Qualidade da Atenção Básica (PMAQ-AB). Foram incluídos municípios que participaram com 80% ou mais de suas equipes em, ao menos, dois ciclos do PMAQ-AB. Foi analisada a correlação entre as taxas de ICSAP padronizadas com a qualidade da APS e demais variáveis. Empregou-se o teste de Spearman entre a variável resposta e as variáveis explicativas numéricas. Foi usado o *generalized equations estimating* como modelo multivariado associando as taxas de ICSAP e as demais variáveis ao longo dos anos.

**RESULTADOS:**

Foram incluídos 3.500 municípios nos modelos. A qualidade da APS (nota do PMAQ-AB) apresentou associação inversa com a variação das taxas de ICSAP. As taxas de internação tiveram queda de -2% ao ano a cada aumento de dez pontos na nota do PMAQ-AB, ajustado pelas demais variáveis. O aumento de uma unidade na variável leitos por mil habitantes impactou em uma elevação de aproximadamente +6,4% nas taxas de ICSAP. Quanto ao porte populacional, municípios maiores tiveram menores taxas de ICSAP. Também se associaram à redução das internações o aumento da cobertura da APS e a menor desigualdade socioeconômica.

**CONCLUSÕES:**

A redução das taxas de ICSAP ao longo do tempo mostrou-se associada com o aumento da qualidade da APS. Além disso, esteve associada com diminuição do número de leitos hospitalares e a municípios com melhores indicadores socioeconômicos.

## INTRODUÇÃO

A atenção primária à saúde (APS) do Brasil, desde 1994, com a criação do Programa de Saúde da Família, teve grande expansão em todo território nacional ^1^ . A partir da criação do Sistema Único de Saúde (SUS), diversas políticas governamentais buscam transformar a APS na principal porta de entrada do sistema, pois existem sólidas evidências de que o seu fortalecimento melhora custos em saúde, impacta em diversos indicadores e contribui na redução das desigualdades em saúde ^2^ .

Avaliar a expansão da APS e a melhoria de sua qualidade é essencial para o planejamento em saúde no país. Um dos pilares de avaliação de qualidade em saúde é a avaliação dos resultados ^3^ . O indicador de internações por condições sensíveis à atenção primária é utilizado em muitos países como um instrumento para avaliar seus resultados ^4^ . Com a criação da lista nacional de internações por condições sensíveis à atenção primária (ICSAP) em 2008, observa-se, no Brasil, um aumento de estudos sobre essa temática, buscando compreender o seu comportamento e sua aplicabilidade ^7 , 8^ .

No Brasil e em suas regiões, séries temporais de ICSAP mostraram queda entre 1999 e 2007. Encontraram também, de forma geral, associação entre essa queda de internações e a expansão da cobertura da APS no território nacional ^9^ . Entretanto, nem sempre essa associação se mostrou verdadeira, especialmente em estudos que analisam dados intraurbanos ou municipais isoladamente ^10 , 11^ . Outros fatores que também mostraram associação com as taxas de ICSAP foram as condições socioeconômicas ^12 , 13^ , os leitos disponíveis no município ^11 , 14^ e a cobertura de planos de saúde ^11^ . Alguns estudos buscaram medir a correlação entre qualidade da APS e taxas de ICSAP por meio do instrumento *Primary Care Assessment Tool* (PCATool) ^13 , 15^ ou do Programa de Melhoria do Acesso e da Qualidade na Atenção Básica (PMAQ-AB) ^12 , 16^ .

O PMAQ-AB, programa de abrangência nacional instituído em 2011, foi realizado ao longo de três ciclos, sendo o último finalizado em 2019. Seu objetivo principal foi induzir a ampliação do acesso e a melhoria da qualidade da APS no país, mas também cumpriu o papel de avaliar as equipes de saúde da família por meio de padrões de qualidade. O PMAQ-AB se destaca pela abrangência e capilaridade do programa, atingindo em seu último ciclo cerca de 39 mil equipes e 96% dos municípios brasileiros ^17 , 18^ . Destaca-se ainda por manter como base para seu processo de avaliação aspectos vinculados aos processos na área assistencial, aos resultados em indicadores e às estruturas nas unidades básicas, considerando o contexto da clássica tríade de Donabedian ^3^ .

Entretanto, ainda faltam estudos nacionais que avaliem a associação entre a variação das taxas de ICSAP e da qualidade da APS por municípios e sua evolução ao longo dos anos, na medida em que a maioria dos estudos avaliam em apenas um município ^13 , 15^ ou em um único ano ^12 , 16^ . Assim, o objetivo deste estudo foi analisar essa associação entre taxas municipais de ICSAP, com a qualidade da APS, variáveis socioeconômicas, demográficas e relacionadas a características locais do sistema de saúde, entre 2010 e 2019.

## MÉTODOS

Trata-se de estudo ecológico, de séries temporais, em que foram analisadas correlações entre as taxas de ICSAP padronizadas nos municípios brasileiros de 2010 a 2019 e a qualidade do cuidado na APS, características de atenção à saúde municipal, condições socioeconômicas e demográficas.

As taxas de internações padronizadas das ICSAP foram calculadas em cada município, com dados provenientes do Sistema de Internação Hospitalar (SIH) de 2010 a 2019. Padronizou-se as taxas por sexo e grupos etários (0–4, 5–19, 20–59, 60–79 anos), utilizando como referência a população brasileira estimada pelo Instituto Brasileiro de Geografia e Estatística (IBGE) em 2014, por ser o ponto médio do período. Foi considerado o ano da alta hospitalar como o ano da internação do paciente e o município de residência para definir o local em que a internação seria contabilizada ^9^ . Foram consideradas as internações por doenças derivadas da décima revisão da Classificação Internacional de Doenças (CID-10) e validadas como condições sensíveis à atenção primária no Brasil composta de 20 grupos diagnósticos divididos em 120 subgrupos de CID-10, excluídos os grupos relacionados ao parto ^7^ .Também foram excluídas do estudo as pessoas com 80 anos ou mais, conforme recomendação do Projeto ICSAP-Brasil ^9^ .

As variáveis do estudo foram apresentadas no Quadro e incluem a qualidade da APS; características dos sistemas de saúde local como: número de leitos por mil habitantes, porcentagem de cobertura de planos de saúde e de cobertura de equipes de saúde da família; características socioeconômicas como Índice Brasileiro de Privações (IBP) e Índice de Desenvolvimento Humano Municipal (IDHM), região e demográficas como sexo e porte populacional (pequeno porte I – até 20.000 hab., pequeno porte II – de 20.001 a 50.000 hab., médio porte – de 50.001 a 100.000 hab., grande porte – de 100.001 a 900.000 hab., metrópole – mais de 900.000 hab.). Os extratos de porte municipal foram definidos segundo estudo de Castro et al. ^12^

**Quadro:** Descrição das variáveis do estudo, segundo tipo de variável, características, fontes de dados e ano de análise da base.

Variáveis	Tipo de variável	Métodos de cálculo	Fonte dos dados	Ano de análise da base
Taxa ICSAP por 10 mil habitantes padronizada	Variável resposta, numérica	Razão anual do número de internações por CID-10 do grupo de ICSAP pela população do município estimada. Multiplicada por 10 mil habitantes e padronizada pela população brasileira em 2014.	SIH-SUS	2010–2019
Qualidade na APS – nota	Variável explicativa, numérica	Média simples das notas das equipes de um mesmo município, em um mesmo ciclo, no processo de certificação do PMAQ-AB. Alguns municípios apresentam notas em apenas dois ciclos.	Banco de dados dos três ciclos do PMAQ-AB. Disponível mediante solicitação ao Ministério da Saúde	2010–2012 (1º ciclo), 2013–2015 (2º ciclo) e 2016–2019 (3º ciclo)
Leitos por mil habitantes	Variável explicativa, numérica	Número de leitos hospitalares conveniados ou contratados pelo SUS, por mil habitantes residentes estimados em determinado município, por ano.	Cadastro Nacional de Estabelecimentos de Saúde (CNES)	2010–2019
Percentual de cobertura de planos de saúde	Variável explicativa, numérica	Razão entre número de vínculos de beneficiários de planos e seguros privados de saúde e a população do município estimado multiplicado por 100.	Agência Nacional de Saúde (ANS), dados e indicadores do setor	2010–2019
Percentual de cobertura de ESF	Variável explicativa, numérica	Cobertura populacional estimada de cada município pelas equipes de saúde da família e de equipes de atenção básica calculada percentualmente.	Portal e-Gestor AB, Informação e Gestão da Atenção Básica, Secretaria de Atenção Primária à Saúde	2010–2019
Porte municipal	Variável explicativa, categórica	Classificação dos municípios segundo sua população: pequeno porte I: até 20.000 hab. pequeno porte II: de 20.001–50.000 hab., médio porte: de 50.001–100.000 hab., grande porte: de 100.001–900.000 hab., metrópole: mais de 900.000 hab.	Estimativa populacional do IBGE disponível no site do Datasus	2010–2019
Sexo	Variável explicativa, categórica nominal	Sexo masculino e feminino de acordo com a população estimada de cada ano.	Estimativa populacional do IBGE disponível no site do Datasus	2010–2019
Região	Variável explicativa, categórica nominal	Região de cada município do estudo.	Dados IBGE	2010–2019
IDHM	Variável explicativa, numérica	Medida nacional composta por três indicadores: renda per capita, escolaridade e expectativa de vida usando dados do Censo de 2010. Medida varia de 0 a 1. Quanto mais próximo de 1, melhor o desenvolvimento humano.	Atlas de Desenvolvimento Humano no Brasil	2010
IBP quintis	Variável explicativa, categórica ordinal	Medida composta de parâmetros de renda, escolaridade e condições do domicílio, usando dados do Censo de 2010. Calculado um escore por cada município brasileiro e desvio-padrão da nota final para agrupar os municípios em cinco categorias ordinais, sendo 5 o índice de maior privação.	Site do Cidacs, Fiocruz (https://cidacs.bahia.fiocruz.br/ibp	2010

ICSAP: Internações por Condições Sensíveis a Atenção Primária. CID-10: Classificação Internacional de Doenças. APS: Atenção Primária à Saúde. SIH: Sistema Internação Hospitalar. PMAQ-AB: Programa de Melhoria do Acesso e da Qualidade da Atenção Básica. IDHM: Índice de Desenvolvimento Humano Municipal. IBP: Índice Brasileiro de Privações. ESF: equipes de saúde da família. IBGE: Instituto Brasileiro de Geografia e Estatística.

A principal variável explicativa, a qualidade da APS, foi calculada pelas notas provenientes da avaliação das equipes de APS do PMAQ-AB, extraídas da base nacional de dados do Programa ( Quadro ). Calculou-se a média simples das notas de equipes de saúde da família de um mesmo município recebidas em cada um dos três ciclos do PMAQ-AB, sendo em cada ciclo uma nota diferente. No primeiro e no segundo ciclo, foi usada nota anterior ao processo de certificação e da ponderação por extratos, para permitir melhores comparações. No terceiro ciclo a nota foi multiplicada por dez, já que foi o único ciclo em que a nota variou de 0,0 a 10,0 e não de 0,0 a 100,0, permitindo assim comparações com os outros ciclos. Para a análise dos dados, as notas do primeiro ciclo do PMAQ-AB foram repetidas em 2010, 2011 e 2012 e correlacionadas com as demais variáveis nos respectivos anos, as do segundo ciclo em 2013, 2014 e 2015 e as do terceiro ciclo com 2016, 2017, 2018 e 2019 ( Quadro ).

Como critério de inclusão para participação no estudo foram selecionados apenas municípios que tiveram ao menos dois ciclos com mais de 80% das equipes de saúde da família e atenção básica participando do estudo do PMAQ-AB. Compuseram o estudo inicialmente cerca de 3.800 municípios. Realizou-se verificação do padrão de distribuição das taxas de ICSAP nesses municípios e exclusão daqueles que, em algum dos anos, tiveram taxas padronizadas menores que 15,0 e maiores que 600,0 por serem valores discrepantes e pela possibilidade de estarem relacionados a erros do sistema de informação e vigilância. Com isso, mais 280 municípios foram excluídos da análise, restando aproximadamente 3.500 municípios, com algumas variações ano a ano, devido à criação de novos municípios no período e dados faltantes sobre internação em determinado ano.

Considerando que cada município esteve nos dez anos de análise (2010 a 2019), e a estratificação das taxas por sexo masculino e feminino, foram cerca de 70 mil unidades de análise (3.500 municípios* dez anos * 2).

Realizou-se a verificação do padrão de distribuição das variáveis. Dado que se observou padrão não normal, foi observada a relação da variável resposta com as variáveis explicativas numéricas por meio da correlação de Spearman ^19^ . A IBP quintis é uma variável categórica ordinal, mas para efeitos de análise foi considerada numérica.

Para avaliar a influência das variáveis nas taxas de ICSAP ao longo do tempo, foi escolhido o modelo de *generalized equations estimating* (GEE) ^20^ . Já que as variáveis tiveram distribuição não normal, com valores contínuos e positivos, assumiu-se o padrão gama. Tendo em vista que as correlações das variáveis IDHM, IBP quintis e Percentual de cobertura de planos de saúde foram fortes, optou-se por fazer três diferentes modelos de GEE para evitar problemas de multicolinearidade.

Para a construção do modelo, ou seja, a seleção de variáveis, foi usado o método *Stepwise* que consiste em adicionar ( *forward* ) e remover variáveis ( *backward)* para definir quais comporão a análise ao final ^21^ . No passo *forward* procedeu-se à análise univariada das variáveis independentes e as que apresentaram um valor-p < 0,20 foram selecionadas para o modelo multivariado. No momento *backward* , retirou-se da análise uma variável por vez que tenha o maior valor-p, repetindo o procedimento até que permaneçam no modelo final somente variáveis significativas. Foi adotado um nível de 5% de significância para o modelo multivariado. Todas as análises foram realizadas no software R, versão 3.6.0, com auxílio do pacote *geepack* .

O estudo foi realizado em conformidade com a Resolução do Conselho Nacional de Saúde (CNS) nº 466, de 12 de dezembro de 2012. As informações do SIH estão disponíveis no site do Datasus, sem a identificação dos pacientes.

## RESULTADOS

A Tabela 1 descreve o padrão de distribuição das taxas de ICSAP e das demais variáveis numéricas ao longo dos dez anos. Observa-se que a variável de qualidade na APS apresentou maior variação das unidades análise (5.670 a 7.004) em função de alguns municípios terem participado de apenas dois ciclos do PMAQ-AB.

**Tabela 1:** Descrição do padrão de distribuição das taxas de ICSAP e variáveis numéricas: qualidade na APS – nota, leitos por mil habitantes, percentual de cobertura de planos de saúde, percentual de cobertura ESF, IDHM, IBP quintis, por número de unidades de análise, média, erro padrão, 1º, 2º, 3º quartil, valor-p, em municípios brasileiros, no período 2010–2019.

Variáveis	Ano	nº válidos	Média	EP	Q1	Q2	Q3	valor-p
Taxa ICSAP por 10 mil habitantes padronizada	2010	6.938	157,72	1,21	82,22	132,48	208,59	< 0,001
	2011	6.968	144,35	1,12	76,18	119,72	188,3	
	2012	6.908	135,19	1,07	70,47	111,25	177,79	
	2013	6.910	127,42	1,01	67,34	105,48	162,4	
	2014	6.948	126,05	1,03	65,07	101,69	161,08	
	2015	6.968	119,35	0,98	61,29	96,73	152,75	
	2016	6.992	114,08	0,95	58,89	91,06	145,11	
	2017	7.028	111,42	0,92	59,25	89,36	139,32	
	2018	6.984	109,9	0,9	58,44	88,96	137,44	
	2019	7.062	107,69	0,86	59,27	87,45	132,54	
Qualidade na APS – nota	2010	5.670	58,95	0,13	53,68	60,11	65,63	< 0,001
	2011	5.686	58,91	0,13	53,58	60,09	65,63	
	2012	5.644	58,99	0,13	53,71	60,13	65,63	
	2013	6.830	53,78	0,12	47,02	54,22	60,8	
	2014	6.870	53,86	0,12	47,15	54,25	60,92	
	2015	6.890	53,9	0,12	47,18	54,32	61,08	
	2016	6.934	59,55	0,14	52,17	60,28	67,2	
	2017	6.974	59,56	0,14	52,17	60,27	67,22	
	2018	6.932	59,54	0,14	52,17	60,3	67,22	
	2019	7.004	59,54	0,14	52,19	60,27	67,18	
IDHM	2010	6.938	0,67	0	0,61	0,67	0,72	1
	2011	6.968	0,67	0	0,61	0,67	0,72	
	2012	6.908	0,67	0	0,61	0,67	0,72	
	2013	6.910	0,67	0	0,61	0,67	0,72	
	2014	6.948	0,67	0	0,61	0,67	0,72	
	2015	6.968	0,67	0	0,61	0,67	0,72	
	2016	6.992	0,67	0	0,61	0,67	0,72	
	2017	7.028	0,67	0	0,61	0,67	0,72	
	2018	6.984	0,67	0	0,61	0,67	0,72	
	2019	7.062	0,67	0	0,61	0,67	0,72	
IBP quintis	2010	6.938	4,01	0,01	3	4	5	0,999
	2011	6.968	4	0,01	3	4	5	
	2012	6.908	4	0,01	3	4	5	
	2013	6.910	4	0,01	3	4	5	
	2014	6.948	4	0,01	3	4	5	
	2015	6.968	3,99	0,01	3	4	5	
	2016	6.992	4	0,01	3	4	5	
	2017	7.028	4	0,01	3	4	5	
	2018	6.984	4	0,01	3	4	5	
	2019	7.062	4	0,01	3	4	5	
Leitos por mil habitantes	2010	6.938	1,58	0,02	0	1,24	2,29	< 0,001
	2011	6.968	1,55	0,02	0	1,21	2,25	
	2012	6.908	1,53	0,02	0	1,18	2,21	
Leitos por mil habitantes	2013	6.910	1,47	0,02	0	1,13	2,16	< 0,001
	2014	6.948	1,47	0,02	0	1,13	2,17	
	2015	6.968	1,45	0,02	0	1,09	2,15	
	2016	6.992	1,41	0,02	0	1,07	2,09	
	2017	7.028	1,4	0,02	0	1,04	2,06	
	2018	6.984	1,37	0,02	0	1,01	2,02	
	2019	7.062	1,31	0,02	0	0,94	1,95	
Percentual de cobertura de planos de saúde	2010	6.938	7,67	0,14	1,03	3,38	9,84	< 0,001
	2011	6.968	8,39	0,2	1,21	3,8	10,73	
	2012	6.908	8,85	0,25	1,36	4,06	11,32	
	2013	6.910	9,06	0,24	1,41	4,23	11,81	
	2014	6.948	9,44	0,18	1,6	4,65	12,84	
	2015	6.968	9,4	0,16	1,59	4,7	12,89	
	2016	6.992	9,11	0,15	1,54	4,64	12,54	
	2017	7.028	8,99	0,13	1,52	4,59	12,57	
	2018	6.984	8,93	0,13	1,55	4,68	12,62	
	2019	7.062	8,83	0,13	1,53	4,64	12,45	
Percentual de cobertura ESF	2010	6.938	0,89	0	0,86	1	1	< 0,001
	2011	6.968	0,85	0	0,78	1	1	
	2012	6.908	0,85	0	0,78	1	1	
	2013	6.910	0,86	0	0,79	1	1	
	2014	6.948	0,89	0	0,87	1	1	
	2015	6.968	0,9	0	0,9	1	1	
	2016	6.992	0,91	0	0,93	1	1	
	2017	7.028	0,9	0	0,92	1	1	
	2018	6.984	0,9	0	0,9	1	1	
	2019	7.062	0,9	0	0,89	1	1	

ICSAP: Internações por Condições Sensíveis a Atenção Primária. APS: Atenção Primária à Saúde; IDHM: Índice de Desenvolvimento Humano Municipal. IBP: Índice Brasileiro de Privações; ESF: equipes de saúde da família. EP: erro padrão. Q1: 1º quartil. Q2: 2º quartil. Q3: 3º quartil.

As médias das taxas de internação declinaram no período, de 157,72 internações por 10 mil habitantes, em 2010, para 107,69/10.000 hab., em 2019. Importante ressaltar que essa não é a taxa do Brasil, mas sim uma média simples entre os municípios do estudo. A média das notas de qualidade da APS construída teve uma queda do primeiro (58,95) para o segundo ciclo (53,80) e a maior média foi a do terceiro ciclo (59,55). A média de número de leitos por mil habitantes também teve queda, saindo de 1,58, em 2010, para 1,31, em 2019. A média do percentual de cobertura de planos de saúde manteve-se entre 7,67% e 9,40% e a média do percentual de cobertura de ESF manteve-se entre 0,85 e 0,91 (85% a 91%). Destaca-se ainda que os dados relacionados aos indicadores de vulnerabilidade (IDHM, IBP quintis) não tiveram variação ao longo dos anos por estarem relacionados ao Censo de 2010 ( Tabela 1 ).

Quando feitas as correlações de Spearman das taxas de ICSAP com as variáveis numéricas ano a ano, observou-se correlação negativa com as notas do indicador de qualidade da APS, com significância estatística nos respectivos municípios, em 2010 e nos anos do terceiro ciclo. Em 2011 e 2012 não houve correlação e, no segundo ciclo, houve correlação positiva ( Tabela 2 ).

**Tabela 2:** Correlações univariadas das taxas de ICSAP com as variáveis numéricas: qualidade na APS – nota, IDHM, IBP quintis, leitos por mil habitantes, percentual de cobertura de planos de saúde, percentual de cobertura ESF, segundo teste de Spearman, coeficiente de correlação e valor-p, em municípios brasileiros, de 2010 a 2019.

Variáveis	Ano	Qualidade na APS – nota ^a^	IDHM ^a^	IBP quintis ^a^	Leitos por mil habitantes ^a^	Percentual de cobertura de planos de saúde ^a^	Percentual de cobertura ESF ^a^
Taxa ICSAP por 10 mil habitantes padronizada	2010	-0,04 (0,008)	0,03 (0,007)	0,00 (0,794)	0,40 (< 0,001)	-0,03 (0,013)	-0,04 (0,001)
	2011	0 (0,744)	0,05 (< 0,001)	-0,01 (0,471)	0,37 (< 0,001)	-0,03 (0,021)	0,00 (0,741)
	2012	0,01 (0,324)	0,08 (< 0,001)	-0,04 (< 0,001)	0,34 (< 0,001)	0,00 (0,796)	-0,01 (0,527)
	2013	0,02 (0,042)	0,09 (< 0,001)	-0,05 (< 0,001)	0,34 (< 0,001)	0,00 (0,693)	-0,02 (0,192)
	2014	0,02 (0,07)	0,10 (< 0,001)	-0,08 (< 0,001)	0,33 (< 0,001)	0,03 (0,007)	-0,02 (0,096)
	2015	0,04 (< 0,001)	0,13 (< 0,001)	-0,10 (< 0,001)	0,34 (< 0,001)	0,05 (< 0,001)	-0,02 (0,161)
	2016	-0,04 (0,001)	0,15 (< 0,001)	-0,12 (< 0,001)	0,32 (< 0,001)	0,07 (< 0,001)	-0,03 (0,021)
	2017	-0,04 (0,001)	0,15 (< 0,001)	-0,12 (< 0,001)	0,34 (< 0,001)	0,07 (< 0,001)	-0,02 (0,179)
	2018	-0,04 (0,001)	0,14 (< 0,001)	-0,11 (< 0,001)	0,32 (< 0,001)	0,07 (< 0,001)	0,00 (0,896)
	2019	-0,04 (0,002)	0,13 (< 0,001)	-0,09 (< 0,001)	0,31 (< 0,001)	0,05 (< 0,001)	-0,01 (0,582)

ICSAP: Internações por Condições Sensíveis a Atenção Primária. APS: Atenção Primária à Saúde. IDHM: Índice de Desenvolvimento Humano Municipal. IBP: Índice Brasileiro de Privações. ESF: equipes de saúde da família.

^a^ Valor das células da tabela: coeficiente de correlação e (valor-p do coeficiente).

A correlação das taxas com indicadores socioeconômicos (IDHM e IBP) ano a ano, de forma univariada, mostrou que as maiores taxas de internação estão em municípios com melhores índices ( Tabela 2 ). Já com relação às variáveis de características municipais, foi observado que a maior força de associação positiva das taxas de ICSAP ocorreu com a variável leitos por mil habitantes (0,31 a 0,40) ( Tabela 2 ).

Os resultados do modelo final que faz a correlação ao longo dos anos por meio do GEE estão apresentados nas Tabelas 3 e 4. Na análise univariada ( Tabela 3 ), todas as variáveis foram significativas (valores-p < 0,05); destaca-se que as variáveis numéricas que apresentaram alterações negativas com a variação das taxas de internação ao longo dos anos, ao aumentar uma unidade foram: qualidade da APS – nota, percentual de coberturas de planos de saúde e percentual de cobertura ESF. As variáveis IDMH e IBP quintis e Leitos por mil habitantes tiveram alterações positivas.

**Tabela 3:** Associações univariadas das taxas ICSAP por 10 mil habitantes padronizada com as variáveis do estudo: qualidade na APS – nota, IDHM, IBP quintis, percentual de cobertura de planos de saúde, leitos por mil habitantes, percentual de cobertura ESF, porte municipal, região, sexo, em municípios brasileiros, de 2010 a 2019.

Variáveis	Análise univariada			

	β	Exp.(β)	Alteração	Valor-p
Qualidade na APS – nota	-0,002	0,998	-0,21%	< 0,001
IDHM (:100) ^a^	0,393	1,482	0,48% ^a^	0,005
IBP quintis	0,018	1,018	1,81%	0,016
log (Percentual de cobertura de planos de saúde) ^b^	-0,097	0,907	-9,26%	< 0,001
Leitos por mil habitantes	0,069	1,071	7,15%	< 0,001
Percentual de cobertura ESF	-0,131	0,877	-12,27%	< 0,001
Porte municipal				
Pequeno porte I	-	1	-	-
Pequeno porte II	0,013	1,013	1,34%	0,573
Médio porte	-0,189	0,828	-17,23%	< 0,001
Grande porte	-0,425	0,654	-34,64%	< 0,001
Metrópole	-0,321	0,725	-27,48%	< 0,001
Região				
Norte	-	1	-	-
Sudeste	-0,008	0,992	-0,83%	0,86
Centro-Oeste	-0,196	0,822	-17,76%	< 0,001
Nordeste	-0,13	0,878	-12,19%	0,002
Sul	0,056	1,057	5,71%	0,174
Sexo				
Feminino	-	1	-	-
Masculino	-0,126	0,881	-11,86%	< 0,001

APS: Atenção Primária à Saúde. IDHM: Índice de Desenvolvimento Humano Municipal. IBP: Índice Brasileiro de Privações. ESF: equipes de saúde da família. β: coeficiente β.

^a^ A alteração foi dividida por 100 para considerar alteração de 0,01 na variável IDHM.

^b^ Valores de percentual cobertura de planos de saúde foram avaliados por log e a todos os percentuais foi adicionado 0,01 por muitas unidades de análise com valor 0.

Na análise multivariada, no primeiro modelo, usando as variáveis do Quadro (exceto IBP quintis e percentual de cobertura de planos de saúde), observou-se que as taxas de internações municipais por ICSAP sofreram um declínio com a melhora da qualidade da APS ao longo dos anos, tendo sido verificado que a cada aumento de 10 pontos na nota da qualidade, havia queda de -2,10% da taxa anual de internação; o aumento percentual de 1% de cobertura de ESF reduziu a internação em -11,81% a cada ano. O aumento do IDHM em 0,10 resultou em redução das taxas de internação em -4,90%. Já o aumento da variável leitos por mil habitantes resultou em elevação nas taxas de internações por condições sensíveis à atenção primária ( Tabela 4 ).

**Tabela 4:** Associações multivariadas das taxas de ICSAP padronizada por 10 mil habitantes, com as variáveis do estudo em três modelos, segundo coeficiente β, Exp. (β), alteração percentual em caso de aumento de uma unidade da variável numérica, valor-p do coeficiente, municípios brasileiros, entre 2010 e 2019.

Variáveis	Modelo 1 - multivariada com IDHM ^c^				Modelo 2 - multivariada com IBP quintis ^d^				Modelo 3 - multivariada log (percentual de cobertura de planos de saúde ^e^			
		
	β	Exp. (β)	Alteração	Valor-p	β	Exp. (β)	Alteração	Valor-p	β	Exp. (β)	Alteração	Valor-p
Qualidade na APS – nota	-0,002	0,998	-0,21%	< 0,001	-0,002	0,998	-0,21%	< 0,001	-0,002	0,998	-0,20%	< 0,001
IDHM (:100) ^a^	-0,668	0,513	-0,49% ^a^	0,001	-	-	-	-	-	-	-	-
IBP quintis	-	-	-	-	0,076	1,079	7,90%	< 0,001	-	-	-	-
Log (percentual de cobertura de planos de saúde) ^b^	-	-	-	-	-	-	-	-	-0,111	0,895	-10,47%	< 0,001
Leitos por mil habitantes	0,062	1,064	6,40%	< 0,001	0,062	1,064	6,39%	< 0,001	0,062	1,064	6,42%	< 0,001
Percentual de cobertura ESF	-0,126	0,882	-11,81%	< 0,001	-0,132	0,877	-12,34%	< 0,001	-0,123	0,884	-11,59%	< 0,001
Porte municipal												
Pequeno porte I	-	1	-	-	-	1	-	-	-	1	-	-
Pequeno porte II	0,039	1,04	4,02%	0,106	0,046	1,048	4,75%	0,059	0,075	1,078	7,77%	0,004
Médio porte	-0,132	0,876	-12,38%	< 0,001	-0,108	0,897	-10,28%	< 0,001	-0,043	0,958	-4,23%	0,157
Grande porte	-0,403	0,668	-33,18%	< 0,001	-0,364	0,695	-30,48%	< 0,001	-0,281	0,755	-24,48%	< 0,001
Metrópole	-0,338	0,714	-28,65%	< 0,001	-0,291	0,748	-25,24%	< 0,001	-0,189	0,828	-17,19%	< 0,001
Região												
Norte	-	1	-	-	-	1	-	-	-	1	-	-
Sudeste	0,001	1,001	0,10%	0,983	0,016	1,016	1,57%	0,73	0,116	1,123	12,33%	0,015
Centro-Oeste	-0,208	0,812	-18,80%	< 0,001	-0,195	0,823	-17,75%	< 0,001	-0,185	0,831	-16,89%	< 0,001
Nordeste	-0,051	0,95	-5,01%	0,2	0,004	1,004	0,35%	0,93	0,116	1,123	12,28%	0,007
Sul	0,113	1,119	11,91%	0,008	0,165	1,18	17,99%	< 0,001	0,243	1,275	27,51%	< 0,001
Sexo												
Feminino	-	1	-	-	-	1	-	-	-	1	-	-
Masculino	-0,117	0,89	-11,05%	< 0,001	-0,117	0,889	-11,05%	< 0,001	-0,123	0,884	-11,56%	< 0,001

APS: Atenção Primária à Saúde. IDHM: Índice de Desenvolvimento Humano Municipal. IBP: Índice Brasileiro de Privações. ESF: equipes de saúde da família.

^a^ A alteração foi dividida por 100 para considerar alteração de 0,01 na variável IDHM.

^b^ Percentual de cobertura de planos de saúde avaliados por log e a todos os valores foi adicionado 0,01 por muitas unidades de análise com valor 0.

^c^ 1º modelo com a variável IDHM e sem IBP quintis e percentual de cobertura de planos de saúde.

^d^ 2º modelo com IBP quintis e sem IDHM e percentual de cobertura de planos de saúde.

^e^ 3º modelo com percentual de cobertura de planos de saúde e sem IDHM e IBP quintis.

No segundo modelo, incluindo a variável IBP quintis e excluindo o IDHM e percentual de cobertura de planos de saúde, o IBP mostrou alteração positiva. Em municípios com quintis de maior vulnerabilidade, observou-se variação positiva nas taxas de internação, mantidos os valores das demais variáveis ( Tabela 4 ).

No terceiro modelo multivariado, excluídos IDHM e IBP quintis e usando o percentual de cobertura de planos de saúde, a associação encontrada com cobertura de planos foi de -11,06% de taxas para cada aumento de 1% de cobertura, ajustados pelas variáveis do modelo ( Tabela 4 ). As demais variáveis numéricas nos modelos 2 e 3 mantiveram suas associações com as taxas de internações semelhantes ao modelo 1.

Entre as variáveis categóricas, as associações negativas foram encontradas com médio, grande porte e metrópole, quando comparados aos municípios de pequeno porte I e mantidas as demais variáveis fixas em todos os modelos ( Tabela 4 ).

Quanto à região, o número das taxas de internação no Centro-Oeste teve variação negativa comparado ao da região Norte nos três modelos em torno de -17%. No Sul, as taxas de internação por ICSAP foram maiores que as da região Norte, variando de 11,91% a 27,51% nos modelos do estudo. No Sudeste e Nordeste observou-se associação positiva apenas no modelo 3. Em relação ao sexo, as taxas de internação no sexo masculino foram menores que no feminino de -11,05% em todos os modelos ( Tabela 4 ).

## DISCUSSÃO

O estudo apontou tendência de redução da média das taxas de ICSAP padronizada no período de dez anos, corroborando achados de estudos de séries temporais em períodos anteriores ^9 , 11^ . Foram usados três modelos na análise multivariada e os fatores que mostraram associação com a redução de internações foram: a melhora da qualidade da APS (medida pelas notas do PMAQ), o aumento de cobertura de equipes de saúde da família (ESF), a diminuição do número de leitos hospitalares vinculados ao SUS e o aumento da cobertura de planos. Além disso, municípios com melhores indicadores socioeconômicos (medidos pelo IBP e pelo IDHM), municípios de médio porte, grande porte e metrópole, ser morador da região Centro-Oeste e ser do sexo masculino associaram-se a menores taxas, enquanto o aumento das taxas de internação foi observado com o aumento do número de leitos por habitantes.

O estudo encontrou associação entre menores taxas de ICSAP em municípios com melhores desempenhos no PMAQ-AB. Esses resultados confirmam estudos anteriores que apontaram redução das taxas de ICSAP ao avaliar os dois primeiros ciclos do PMAQ-AB ^12 , 16^ , o aumento de adesão de equipes ao longo dos três ciclos ^22^ , ou que compararam municípios que aderiram no PMAQ-AB, com aqueles que não aderiram ^23^ . Este estudo avança ao apontar que, no período de dez anos, mesmo entre os municípios que participaram com ao menos 80% das suas equipes, o aumento de dez pontos nas notas no decorrer dos ciclos do PMAQ-AB está associado à redução das taxas em -2% ao ano, ajustado pelas outras variáveis.

A associação com qualidade da APS, entretanto, não foi encontrada por Gonçalves et al. ^15^ em estudo de coorte em Porto Alegre e nem por Mendonça et al. ^13^ em estudo em Belo Horizonte. Ambas as pesquisas usaram o PCATool como indicador de qualidade e avaliaram série temporal em um município isoladamente.

Ao relacionar a queda das taxas de ICSAP com a cobertura da ESF, municípios que tiveram aumento de 1% nos índices tiveram reduções das taxas no período da ordem de -11,5 a -12,5%. Essa correlação também foi observada em estudos anteriores, os quais consideraram que o aumento de cobertura da ESF seria um fator explicativo para essa melhora ^9 , 24^ .

Na análise em um ano de forma univariada, foi observado que maiores taxas de cobertura ESF não estão associadas a menores taxas de internação. Diferentemente da ampliação de cobertura ao longo dos anos, pois esse movimento sim pode estar relacionado a um aumento real de investimentos, de fortalecimento e expansão da APS ^11 , 25^ .

Quanto às variáveis socioeconômicas, identificou-se que o aumento do IDHM, associado a melhores condições de vida, resultou na queda das taxas de internação. O Índice Brasileiro de Privação (IBP) também identificou correlação nos quintis de maior vulnerabilidade, maior pobreza e aumento de internação. Esses achados são convergentes com outros estudos que correlacionaram vulnerabilidade e internação, e que locais com maior privação apresentam piores indicadores em saúde ^12 , 13 , 26^ .

A variável leitos por mil habitantes apresentou a maior correlação em todos os anos na análise univariada e manteve associação positiva nos três modelos multivariados investigados. O aumento no indicador leitos por mil habitantes impactou em uma elevação nas taxas de ICSAP, ajustado pelas outras variáveis. Essa associação já foi encontrada em outros estudos e pode estar relacionada com maior demanda por internação em locais com maior disponibilidade de leitos ^12 , 23 , 27 , 28^ .

O estudo identificou que o aumento da cobertura de planos de saúde resultou na queda das internações por ICSAP. Os achados de diminuição de ICSAP relacionados ao aumento de cobertura de planos privados de saúde também já foram descritos ^12^ . Possivelmente esse indicador reflete melhores condições socioeconômicas desses municípios. Pode ainda ter relação com a ausência de dados de internação no setor não conveniado ao SUS nos cálculos das taxas de ICSAP.

Quanto às características demográficas foi observado nos três modelos uma diminuição das taxas por municípios quando estes são de médio, grande porte e metrópole em relação à comparação com municípios de pequeno porte I. Esse achado é condizente com os resultados do estudo de Carmo ^28^ (2016), que aponta que hospitais com até 49 leitos, considerados de pequeno porte, concentravam 35% das ICSAP do país em 2015. A autora salienta que esse perfil resulta da baixa complexidade dos hospitais, pequena diversidade nas especialidades atendidas, e que, em Minas Gerais, quase metade deles estava localizada em cidades de até 10 mil habitantes e que esses hospitais têm maior demanda e pressão por internação ^3 , 28^ .

O sexo masculino apresenta menores taxas de internação. Estudos apontam que as mulheres procuram mais os serviços de saúde e, em geral, apresentam melhor percepção de sinais e sintomas de doenças e procuram com maior frequência os serviços de saúde ^29^ , esta última podendo resultar em maior oportunidade de tratamentos e de internações.

Os achados são importantes para reforçar a necessidade de investimentos em qualidade da APS e em programas avaliativos, ante a descontinuidade do PMAQ-AB. Novos estudos de seguimento em locais que investiram em qualidade da APS são importantes, bem como estudar o impacto da variação ao longo do tempo de condições socioeconômicas. Também são necessários estudos investigando a correlação de municípios menores e maior demanda de internação nessas localidades.

### Limites

Trata-se de estudo ecológico podendo existir vieses de associação pela concorrência de muitos e imbricados fatores explicativos. O uso de taxas de ICSAP demanda cuidados com suas interpretações, pois podem não refletir apenas o desempenho da APS. É um indicador que diz respeito à organização dos sistemas de saúde e sofre influências de diversos outros fatores ^11^ . Pode haver ainda problemas relacionados à base de dados de internação por não incluir dados de hospitais não conveniados ao SUS, o que pode interferir na análise e na interpretação, especialmente em cidades com maior cobertura de planos de saúde ^12 , 23^ . Ainda assim, o SIH é uma fonte de informação de significativa confiabilidade, permitindo relevantes análises epidemiológicas sobre a morbidade hospitalar ^9^ . As variáveis IBP e IDHM são baseadas no Censo de 2010 e, portanto, não conseguem medir a variação socioeconômica dos municípios no período, entretanto permaneceram no modelo por medirem desigualdades sociais. Destaca-se também que a adesão ao PMAQ-AB pelos municípios e equipes a serem avaliadas era voluntária, podendo haver viés de seleção das equipes, especialmente no primeiro ciclo.

## CONCLUSÕES

O estudo aponta que melhores resultados na qualidade da APS no Brasil resultam em redução das taxas de ICSAP, reafirmando a necessidade de se avançar no país para além da expansão de cobertura em atenção básica. Também se observou que locais com maiores privações socioeconômicas também tiveram taxas mais altas de internação no estudo, confirmando que a desigualdade social se relaciona de forma importante com resultados em saúde. A disponibilidade de leitos por mil habitantes e, em especial, em municípios com menos de 20 mil habitantes, parece ter relação com mais internações.

O estudo também aponta a necessidade de investir em programas avaliativos considerando a tríade de Donabedian em escala nacional e que consigam ter um papel de indução de melhorias na APS e de indicações de que rumos seguir em direção a um sistema de saúde mais efetivo e resolutivo.
